# Herb Formula (GCis) Prevents Pulmonary Infection Secondary to Intracerebral Hemorrhage by Enhancing Peripheral Immunity and Intestinal Mucosal Immune Barrier

**DOI:** 10.3389/fphar.2022.888684

**Published:** 2022-05-23

**Authors:** Yulu Miao, Bin Wang, Jing Hu, Hanyu Zhang, Xiaojin Li, Yingying Huang, Pengwei Zhuang, Yanjun Zhang

**Affiliations:** ^1^ Chinese Materia Medica College, Tianjin University of Traditional Chinese Medicine, Tianjin, China; ^2^ College of Pharmacy, Anhui University of Chinese Medicine and Anhui Academy of Chinese Medicine, Hefei, China; ^3^ Tianjin State Key Laboratory of Modern Chinese Medicine, Tianjin University of Traditional Chinese Medicine, Tianjin, China

**Keywords:** intracerebral hemorrhage, immunosuppression, intestinal mucosal immune barrier, formula GCis, transcriptomics

## Abstract

Lung infection is a common complication induced by stroke and seriously affects the prognosis and life quality of patients. However, effective therapeutic strategies are still lacking. In the present study, the herb formula GCis was confirmed to prevent pulmonary infection induced by intracerebral hemorrhage (ICH). The animal model of lung infection induced by ICH, GCis (Ginseng Radix et Rhizoma, Aconiti Lateralis Radix Praeparata, and Cistanches Herba) was orally administrated every day for 7 days. Lung microbial biomass and pathological results showed that the GCis formula pretreatment significantly reduced lung bacterial biomass and alleviated pathological abnormalities. These results indicated that the GCis formula has a clear pharmacological effect on preventing lung infection induced by ICH. Immunosuppression induced by ICH seemed to be the main mechanism of lung infection. Our results showed that the spleen and thymus indexes, WBC, and LY% contents were significantly increased in the GCis formula group. Moreover, bone marrow cells were further analyzed by transcriptome sequencing, and GO and KEGG enrichment analysis results showed that immune function was the main pathway enriched by differential genes after GCis formula intervention. More importantly, our results showed that GCis pretreatment had no significant effect on the mRNA expression of IL-1β, IL-6, and TNF-α in the brain. These results indicated that the GCis formula could enhance immunity after ICH. The intestinal barrier function was further investigated in the present study, considering the origin of the source of infection. Our results showed that the mRNA expressions of intestinal ZO-1, SIgA, and MUC2 were significantly increased, villi structure was intact, inflammatory cell infiltration was reduced, and goblet cell number was increased after GCis formula treatment. These results suggest that the GCis formula can enhance the intestinal mucosal immune barrier. This study provides a herb formula (GCis) that could enhance peripheral immunity and intestinal mucosal immune barrier to prevent pulmonary infection induced by ICH. It would be beneficial in the prevention of severe clinical infections.

## Introduction

Stroke is a common central nervous system disease with a high disability rate and mortality, which can be divided into ischemic stroke and hemorrhagic stroke. ICH accounts for 10%–17% of all strokes, and the mortality rate of patients is as high as 35%–52% (Hazeldine and Lord, 2015; Kopp et al., 2017; Li and Liu, 2017). Lung infection is a common complication induced by ICH and seriously affects the prognosis and life quality of patients. The total infection rate of stroke-related infections (pneumonia and urinary tract infection) is 23%–65%, and about 30% of deaths are related to them (Schaeffer, 2015; Shim, 2016). Therefore, poststroke infectious complications were reported to be a major reason for the readmission and death of stroke patients and associated with poor outcomes. Currently, antibiotics are still recommended as the clinical treatment of infection after ICH. Although the infection can be controlled, it is related to various side effects on the treatment and prognosis of the primary disease (Shi et al., 2018; Boning and Xiaojiaoyang, 2020). In addition, prophylactic use of antibiotics and β-blockers propranolol does not reduce the incidence of lung infection, and neurological function and prognosis do not improve (Westendorp et al., 2015). Hence, the development of a new drug is still very urgent and imperative for preventing lung infection induced by ICH.

The formulas of Traditional Chinese medicine (TCM), as an essential alternative and complementary medicine, have been widely utilized to recover the harmony disturbed in diseases through multi-target synergistic functions of its corresponding components (Wang et al., 2012; Zhang et al., 2015). Considerably, increasing clinical evidence demonstrates the desirable efficiency of the prescription of TCM in lung infection induced by ICH by alleviating the symptoms and improving lung function and quantity of life (Haifeng et al., 2015). GCis formula consists of three crude herbs, namely, Renshen (*Ginseng Radix et Rhizoma*), Fuzi (*Aconiti Lateralis Radix Praeparata*), and Rou Congrong (*Cistanches Herba*) in a specific ratio of 1:1:1. Ginseng invigorates the spleen and lungs, greatly invigorating the vitality; Aconite seed tonifies kidney Yang; Cistanche burns fire and helps Yang. This prescription is suitable for the stroke of Qi Xu, Fei Xu, and Qi Xue Liang Xu (Yu, 2016). However, the mechanisms of the TCM theory-based synthetic effects of Qi Xu, Fei Xu, and Qi Xue Liang Xu in GCis are not reported.

Clinical studies have found that patients with ICH injury showed impaired immune function, including a rapid and sustained decline of cellular immune function, spleen, thymus, and lymph node atrophy (Prüss et al., 2017; Wong et al., 2017). In addition, there is a link between CNS, gut, and immune strictly, and regulated control of gut integrity is particularly important for host antibacterial immune defense (Stanley et al., 2018). In light of these, the regulation of immune function might be an effective modality to treat infection after ICH injury.

In the present work, with the animal model of lung infection induced by ICH, we assessed the lung bacterial biomass, and then the transcriptome of ileum and bone marrow were analyzed using RNA-Seq technology to elucidate the underlying mechanisms of GCis. Our study will highlight the synthetical prevention of lung infection induced by ICH superiority of the GCis formula and provide a foundation for future analyses of this and other TCM.

## Materials and Methods

### Extraction of GCis

GCis formulas were prepared with Renshen (White Ginseng), Fuzi (Hei Shunpian), and Rou Congrong combination (1:1:1). The herbs were acquired from Sinopharm Group Beijing Huamiao Pharmaceutical Co., Ltd. (Beijing, China). The combined extraction liquid was soaked with 10-time distilled water for 30 min, then microboiled for 1 h twice, and mixed with the twice filtrate. Finally, the mixture was concentrated and dried to produce the GCis.

### Animal Experimental Design

Male C57BL/6J mice (7–8 weeks old, 20–24 g, Experimental Animal Center, Vital River Laboratory Animal Technology Co., Ltd., Beijing, China) were housed in cages with a temperature of 22 ± 2°C and humidity of 40 ± 5%, under a 12 h light/dark cycle. Standard mouse chow and water were provided ad libitum. The mice adapted for 7 days and were randomly divided into four groups: Control, Model, GCis-L, and GCis-H (*n* = 15 per group).

After the 7-day habituation period, except for mice in the control group, the collagenase IV-S (St. Louis, MO, United States) was performed on all animals to produce ICH models. Following intraperitoneal injection of 40 mg/kg pentobarbital, mice were placed in a stereotaxic frame, shaved, and swabbed the incision area with 70% ethanol. A 30-gauge needle was inserted through a burr hole on the skull into the striatum (stereotaxic coordinates: 0.5 mm anterior to the bregma, 2.3 mm lateral to the midline, and 3.7 mm below the skull). ICH was induced by microinfusion pump-mediated injection of 0.03U collagenase type IV-S in 0.5 μL saline at a constant rate of 0.2 μL/min. The needle was slowly removed after an additional 5 min delay to prevent backflow. Body temperature was maintained at 37°C with a rectal probe and a heating blanket until the mice woke up.

Three days before ICH surgery, the mice in treatment groups were intragastrically administered with GCis once daily for 3 days: the GCis-L group received 1.3 g/kg/d, the GCis-H group received 3.9 g/kg/d, and the sham-operated and ICH model groups received distilled water *via* gavage at the dose of 10 ml/kg. On the day of surgery and after the operation, the mice continue to receive drugs or distilled water for 7 days.

On day 7, the mice were anesthetized intraperitoneally, the viscera (brain, lung, thymus, spleen, bone marrow, and ileum) were rapidly taken out and stored at a −80°C refrigerator, the viscera and body weights were calculated as an index of viscus, and brain edema was measured based on brain weight.

### Neurobehavioral Tests

Examinations of neurological deficits of the experimental animals were performed 7 days after surgery. The neurological function was blindly scored on the wire hang test, beam walking test, and elevated body swing test (EBST) as previously described (Wu et al., 2009). The wire hang and beam walking tests were graded from 0 to 5 (0, the worst; 5, the best). Greater scores indicated better neurological functions. EBST was calculated as the percentage of right-biased swings (R) in the total swings recorded (20 times, sum of left- and right-biased swings), R/(L + R).

### Measurement of ICH Area

Twenty-four hours after the last administration, mice were anesthetized and the brain tissue was harvested quickly except for the olfactory bulb, cerebellum, and lower brainstem. Afterward, the brain was dissected into coronal slices (2 mm thick, *n* = 6). Generally, the slices were digitized and analyzed with ImageJ software, and the hemorrhagic injury volume was presented as a percentage (%) of (total hematoma volume/total brain volume) × 100.

### Histological Examination

Lung and ileum tissue were harvested and fixed in 4% (v/v) buffered paraformaldehyde, then embedded in paraffin, sectioned at 4 μm thickness, applied to glass slides, and stained with hematoxylin and eosin (H&E) solution. Afterward, the goblet cell was observed by Alcian blue/periodic acid-Schiff (AB/PAS) straining. HE and AB/PAS-stained images were acquired with an upright microscope in ×100 and ×200 magnification (Leica Microsystems, Wetzlar, Germany).

### Quantification of Lung Biomass

By using the EasyPure^®^ Genomic DNA Kit (Transgen, Beijing, China), total DNA was isolated from lung tissue. qPCR was conducted on samples to determine the relative amount of bacteria using published bacteria-specific primer sequences (Shim, 2016; Bronchard et al., 2004) for SYBR Green reagent (Bio-rad) on a CFX96Touch RT-PCR system (Bio-rad). The 16Sr RNA/Rpp30 value was calculated by the 2^−dCt^ method to indicate the bacterial load of lung tissue.

### Assessment of Intestinal Permeability

The lactulose/mannitol (L/M) test is widely used to evaluate intestinal permeability in humans and animals, including mice. Animals fasted for 8 h, gavaged with 0.5 ml lactulose and mannitol mixture solution (containing 60 mg/ml fructose and 40 mg/ml mannitol), and housed in metabolic cages individually. Urine samples were collected after 12 h. Lactulose and mannitol concentrations for each urine sample were quantified by UPLC/MS, as previously described (Gervasoni et al., 2018; Xu et al., 2015). Final data were presented as a ratio of lactulose and mannitol.

### Measurement of SIgA in Intestinal Mucus

Intestinal mucus from the duodenum to terminal ileum was collected with a smooth pincette and transferred to an Eppendorf tube, homogenized in 0.5 ml cold PBS, and then centrifuged at 4°C at 3,000 r/min for 10 min. The supernatant was collected, and the content of SIgA was measured according to the manufacturer’s protocol. Then, the protein content of the supernatant was determined by the BCA method. The SIgA level was expressed as nanograms per milligram of total protein.

### UPLC-Q-TOF/MS Determination Content of Neurotransmitter

Ileum tissue samples were weighed to 30 mg, and 200 μL water MP homogenate was added, followed by vortexing for 60 s. Then, an 800 μL methanol acetonitrile solution (1:1, V/V) was added, followed by vortexing for 60 s , at low-temperature ultrasonic for 30 min, twice. Precipitated protein was placed at −20°C for 1 h, 14,000 rpm. Finally, after centrifuging at 4°C for 20 min, the supernatant was freeze-dried, and samples were stored at −80°C. While plasma samples were slowly dissolved at 4°C, 100 μL of each sample was taken, and 400 μL of pre-cooled methanol acetonitrile solution (1:1, V/V) was added to the plasma samples, followed by vortexing for 60 s, precipitation of protein, drying as above, and preservation of samples at −80°C.

Samples were analyzed by UPLC-Q-TOF/MS (Agilent 1290 Infinity LC/AB SCIEX). The peak area and retention time were extracted by Multiquant software, the retention time was corrected with neurotransmitter standard, and metabolites were identified. Information on all ion pairs of neurotransmitters is shown in Table 1.

**TABLE 1:** Information on all ion pairs of neurotransmitters.

Component name	Analyte mass range	Analyte retention time (min)
Acetylcholine	146.1/60.1	3.08
Norepinephrine	170.3/107.1	7.96
γ-Amino-butyric acid	104.0/87.1	8.49
Serotonin	177.0/160.1	5.92
5-HIAA	192.0/146.1	1.27

### RNA-Seq Sample Preparation and Analysis

Total RNA of ileum and bone marrow (*n* = 5) were extracted using TRIzol^®^ reagent (Invitrogen, Thermo Fisher Scientific Inc., USA). The integrity of the 28 and 18 s ribosomal RNA was determined on a 1% agarose gel to assess the degradation degree of RNA. While the concentration and purity of the isolated RNA were estimated by NanoDrop spectrophotometric measurements (ND-2000, NanoDrop, Thermo Fisher Scientific Inc.), the RNA integrity (RIN) was measured in the Agilent 2100 bioanalyzer (Agilent Technologies Inc., Germany). The RNA-Seq was performed at Applied Protein Technology (Shanghai, China). The directional cDNA libraries were prepared, the constructed library was qualified and quantified using the Qubit 2.0 fluorometer and the Agilent 2100 bioanalyzer, and the Illumina HiSeq platform was used for sequencing. The raw reads obtained from HiSeq sequencing are processed to obtain high-quality sequences (Clean Reads) by removing low-quality sequences and removing adaptor contamination. All subsequent analyses are based on Clean Reads. The gene expression level was measured in units of fragments per kilobase of transcript sequence per millions of base pairs sequenced (FPKM). *p*-value <0.05 and |log_2_ foldchange|>1 were used for selecting differentially expressed genes (DEGs). The volcano plot and cluster heatmap were performed on the normalized gene expression data to visualize the resulting expression intensity values of the ileum transcripts (Xu et al., 2019).

In our study, the up-DEGs and down-DEGs in Mod *vs*. Sham and down- or upregulation in GCis *vs*. Mod were filtered out as reverse-regulated DEGs. The STRING tool (STRING; https://string-db.org/) was used to generate PPIs among the reverse-regulated DEGs. Then, Cytoscape was used to visualize the network. To explore gene function and regulatory networks of reverse-regulated DEGs, the enrichment analysis of Gene Ontology (GO) terms and Kyoto Encyclopedia of Genes and Genomes (KEGG) metabolic pathways mapping were conducted.

### Quantitative Reverse Transcription-PCR

Brain, lung, and ileum tissues were homogenized in RNA lysis buffer. Total RNA was extracted using the Eastep total RNA extraction kit (Promega, Beijing). Equal amounts of RNA were reversely transcribed into cDNA with FastQuant RT kit (with gDNase) (Tiangen, Beijing) in accordance with the manufacturer’s instructions. Relative gene expression was determined using specific quantitative primers (see Table 1). Polymerase chain reactions were performed on a CFX96 touch system (Bio-Rad, United States) with SuperReal PreMix Plus (SYBR Green) kit (Tiangen, Beijing). The mRNA expression levels were normalized to GAPDH using the ^ΔΔ^Ct relative to control groups.

### Statistical Analysis

For comparison of multiple groups, one-way analysis of variance (ANOVA) followed by the least-significant difference method was applied (GraphPad Prism 7 Software, Inc., La Jolla, CA, United States). *p*-value < 0.05 was considered statistically significant. Data were analyzed by SPSS 22.0 software (International Business Machines Corp, Armonk, NY, United States, RRID: SCR_019096). The achieved data were expressed as the mean ± SEM.

## Results

### GCis Formula Pretreatment Ameliorated Pulmonary Infection Induced by ICH

#### GCis Formula Decreased Area of ICH and Improved Neurological Function Scores

**FIGURE 1:**
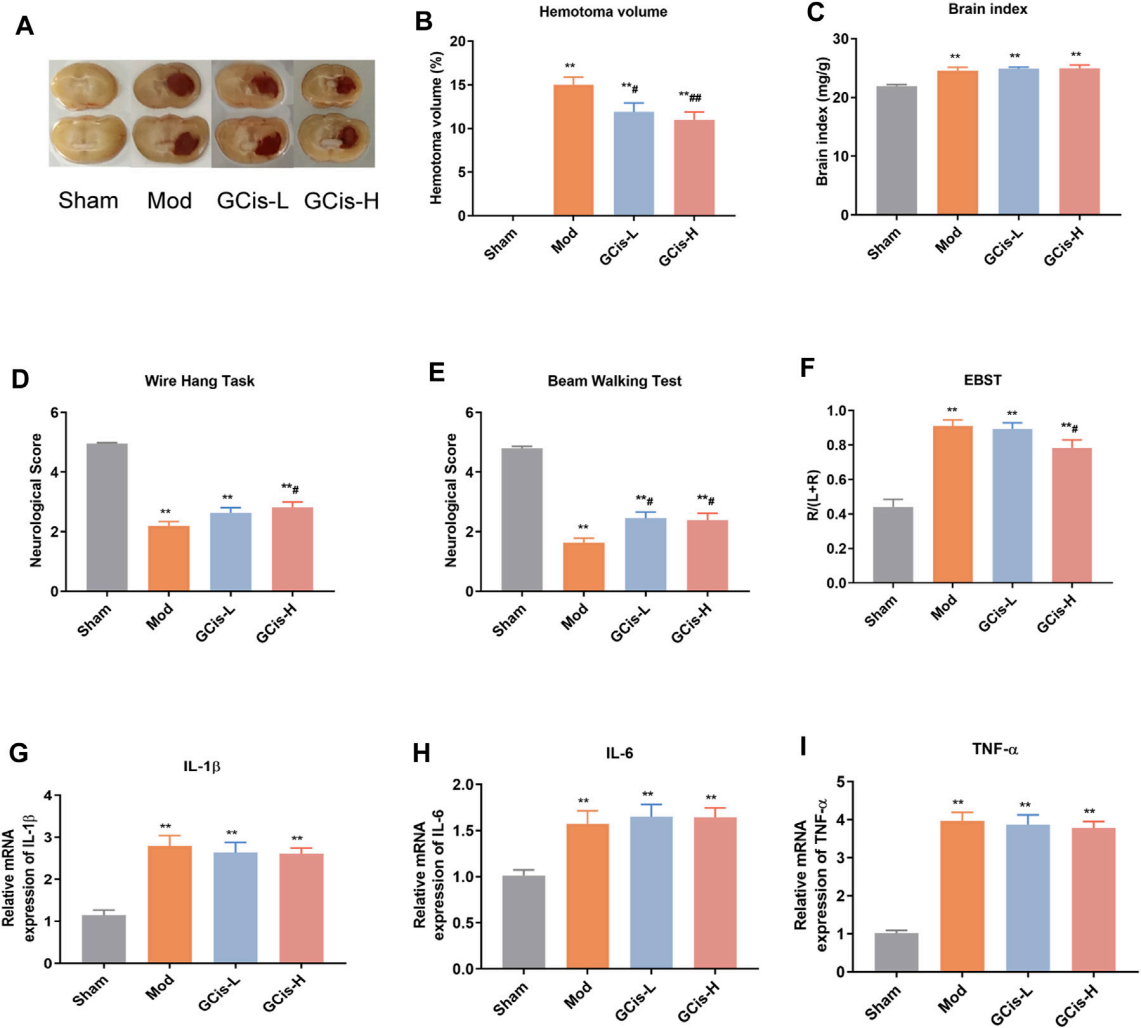
GCis formula decreased area of ICH and improved neurological function scores. **(A)** Area of ICH. **(B)** hematoma volume of ICH, **(C)** brain index of ICH, **(D)** beam walking test, **(E)** wire hang test, **(F)** EBST, **(G)** IL-1β, **(H)** IL-6, and **(I)** TNF-a in the brain. Data are shown as mean ± SEM. **p < 0.01 compared with the sham group; ^#^ *p* < 0.05 and ^##^ *p* < 0.01 compared with the model group.

We observed a hematoma volume (Figures 1A,B), and brain index (Figure 1C) were significantly increased compared to the control group. In contrast, the hematoma volume was substantially ameliorated with GCis formula treatment (*p* < 0.01) and the brain index of GCis formula showed no significant difference compared to the model group. Furthermore, as shown in Figures 1D–F, neurobehavioral tests also presented that GCis formula treatment markedly increased the neurological score of the wire hang test, beam walking test (*p* < 0.05), and decreased the neurological score of EBST. A memorably higher level of IL-1β (Figure 1G), IL-6 (Figure 1H), and TNF-α (Figure 1I) were observed in ICH mice (*p* < 0.01), but no significance was observed with GCis formula treatment.

#### GCis Formula Pretreatment Ameliorated Pulmonary Infection

**FIGURE 2:**
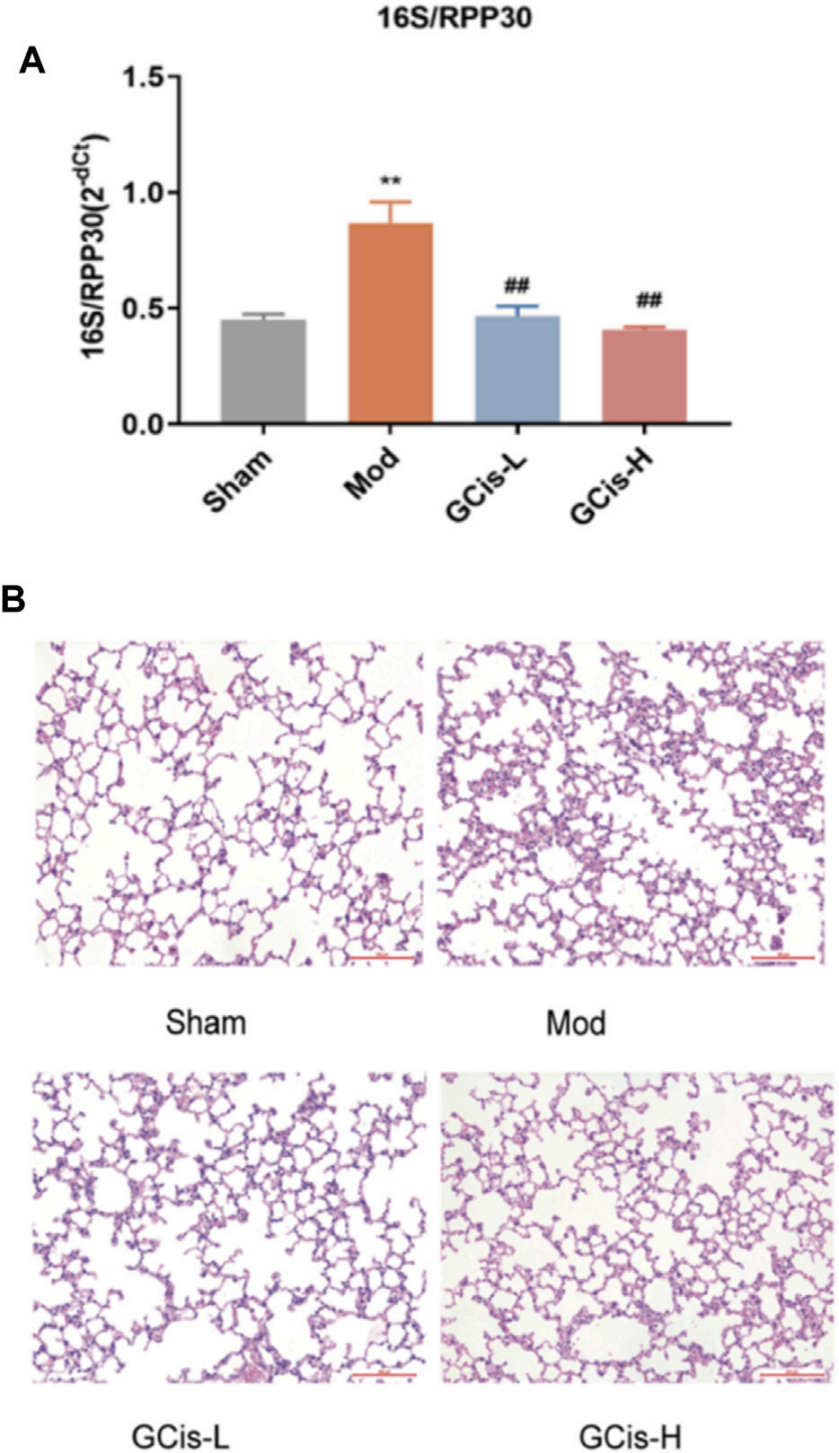
GCis formula pretreatment ameliorated pulmonary infection. **(A)** Q-PCR 16S/RPP30 was measured by qRT-PCR. **(B)** Typical histological images of H&E-stained lung tissues (magnification ×100). Data are shown as mean ± SEM. **p < 0.01 compared with the SHAM group; ^##^ *p* < 0.01 compared with the model group.

16S/RPP30 was used to evaluate the relative content of intestinal flora in the lung tissue (Figure 2A). The relative content of intestinal flora was significantly increased compared with the control group. However, it was substantially ameliorated with GCis formula treatment (*p* < 0.01). H&E staining was used to evaluate the inflammatory changes in the lung tissue. Extensive infiltration of inflammatory cells into peribronchial and perivascular regions was observed in ICH mice with significantly increased inflammatory scores, while they were significantly decreased with GCis treatment (Figure 2B).

### GCis Formula Preconditioning Enhanced Peripheral Immunity in the Lung Infection Mice Induced by ICH

**FIGURE 3:**
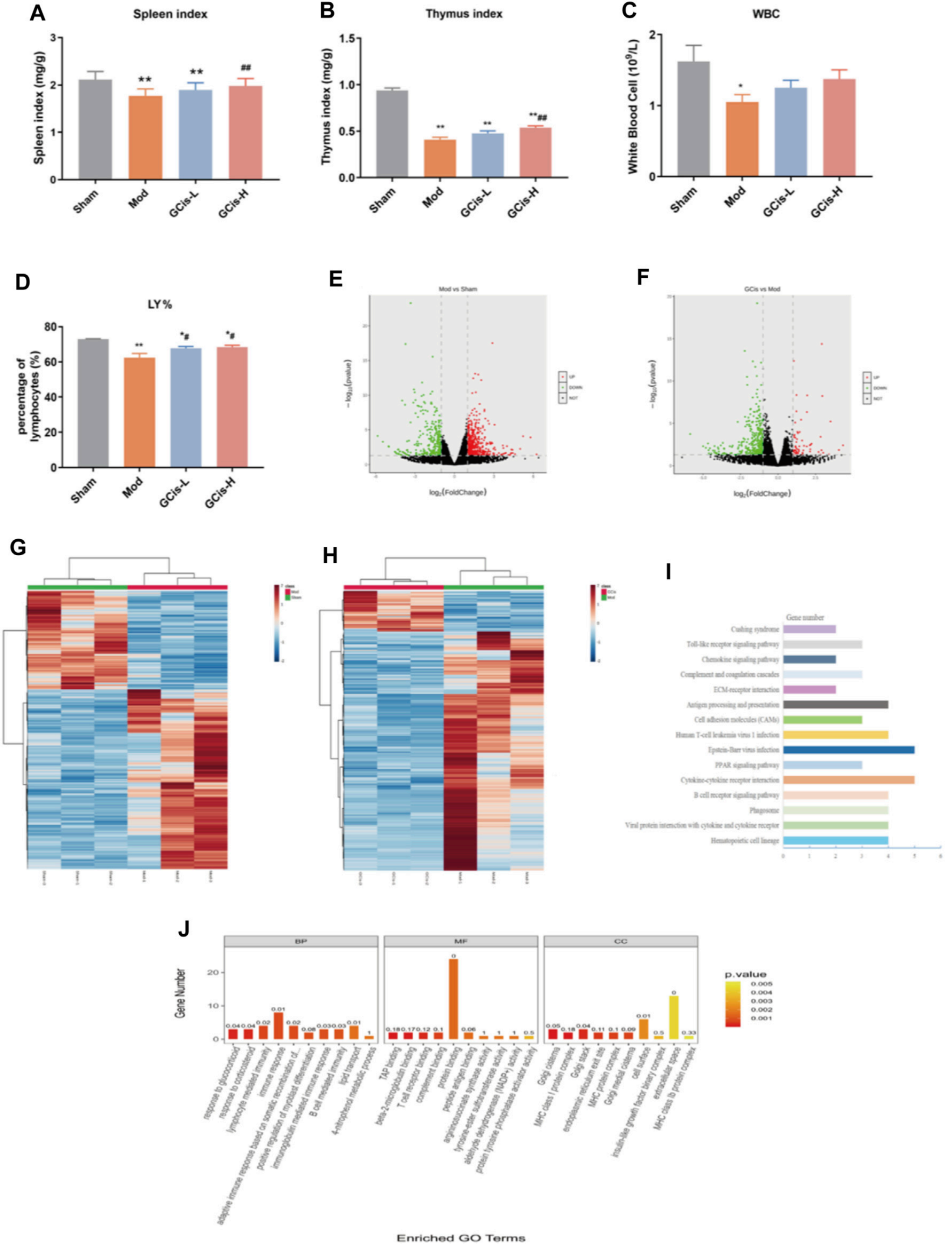
GCis formula preconditioning enhanced peripheral immunity in the lung infection mice. **(A)** Spleen index, **(B)** thymus index, **(C)** white blood cell, and **(D)** LY%. The upregulated (red) or downregulated (blue) genes **(E)**. **(G)** Sham group *vs*. model group **(F)**. **(H)** GCis group *vs*. model group. Bioinformatics analysis (GO and KEGG annotation) of 61 DEGs. **(I)** KEGG analysis of GCis 61 DEGs. **(J)** GO analysis of GCis 61 DEGs (data are shown as mean **±** SEM. *p < 0.05 and **p < 0.01 compared with the sham group; ^#^ *p* < 0.05 and ^##^ *p* < 0.01 compared with the model group).

On day 7, the spleen, thymus index, white blood cell (WBC), and lymphocyte (LY%) were significantly reduced within the groups of the GCis formula in comparison with the control group (*p* < 0.01) (Figures 3A–D). After 1 week of treatments, the spleen, thymus index, and LY% of the GCis formula group (*p* < 0.01) were significantly increased than the model group, suggesting that the GCis formula can improve the peripheral immunity in the lung infection mice induced by ICH.

DEGs were screened out by *p*-value ≤ 0.05 and |log_2_ foldchange|≥1. The volcano plot showed the relative changes in gene levels (Figures 3E,F). In the model *vs*. sham group, 855 DEGs were identified (552 upregulation and 303 downregulation). In the GCis formula *vs*. model group, 420 DEGs were identified (61 upregulation and 359 downregulation). A hierarchical cluster was used to obtain the sample clustering results (Figures 3G,H). There were significant differences in gene expression between the model and sham groups, GCis and model group, and high similarity in gene expression between the same group.

Based on 61 GCis formula DEGs, the GO and KEGG pathway enrichment was analyzed (Figures 3I,J). The enriched pathways were mainly related to immune response, including synthesis and release of immune cells, B-cell-mediated immunity, lymphocyte-mediated immunity, adaptive immune response based on somatic recombination of immune, and Toll-like receptor signaling pathway.

#### Validation of DEGs

**FIGURE 4:**
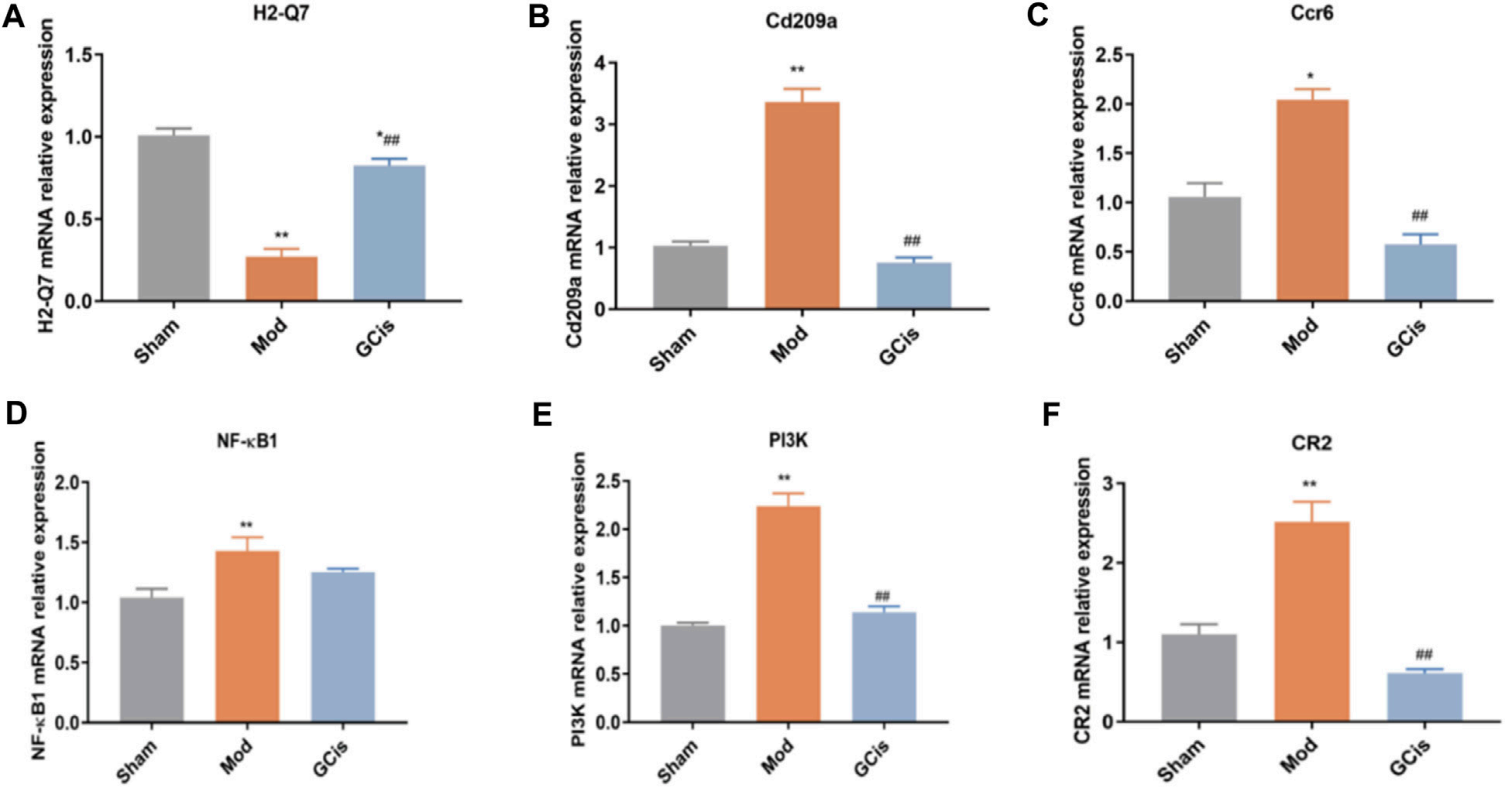
Validation of H2-Q7 **(A)**, Cd209a **(B)**, Ccr6 **(C)**, NF-κB1**(D)**, PI3K **(E)**, and CR2 **(F)**. Data are shown as mean ± SEM. *n* = 5, *p < 0.05 and **p < 0.01 compared with the sham group; ^##^ *p* < 0.01 compared with the model group.

QRT-PCR was executed to further validate the candidate DEGs containing H2-Q7, Cd209a, Ccr6, NF-κB1, PI3K, and CR2. As displayed in Figure 4A, GCis formula treatment upregulated H2-Q7 significantly compared to the model group (*p* < 0.01). Figures 4B,C show that the GCis formula treatment downregulated Cd209a and Ccr6 significantly compared to the model group (*p* < 0.01). As displayed in Figure 4D, the GCis formula treatment showed no significance with NF-κB1 compared to the model group. Figures 4E,F show that the GCis formula treatment downregulated PI3K and CR2 significantly compared to the model group (*p* < 0.01).

### GCis Formula Pretreatment Improved Intestinal Barrier Function in the Lung Infection Mice Induced by ICH

#### GCis Formula Enhanced the Intestinal Barrier Function

**FIGURE 5:**
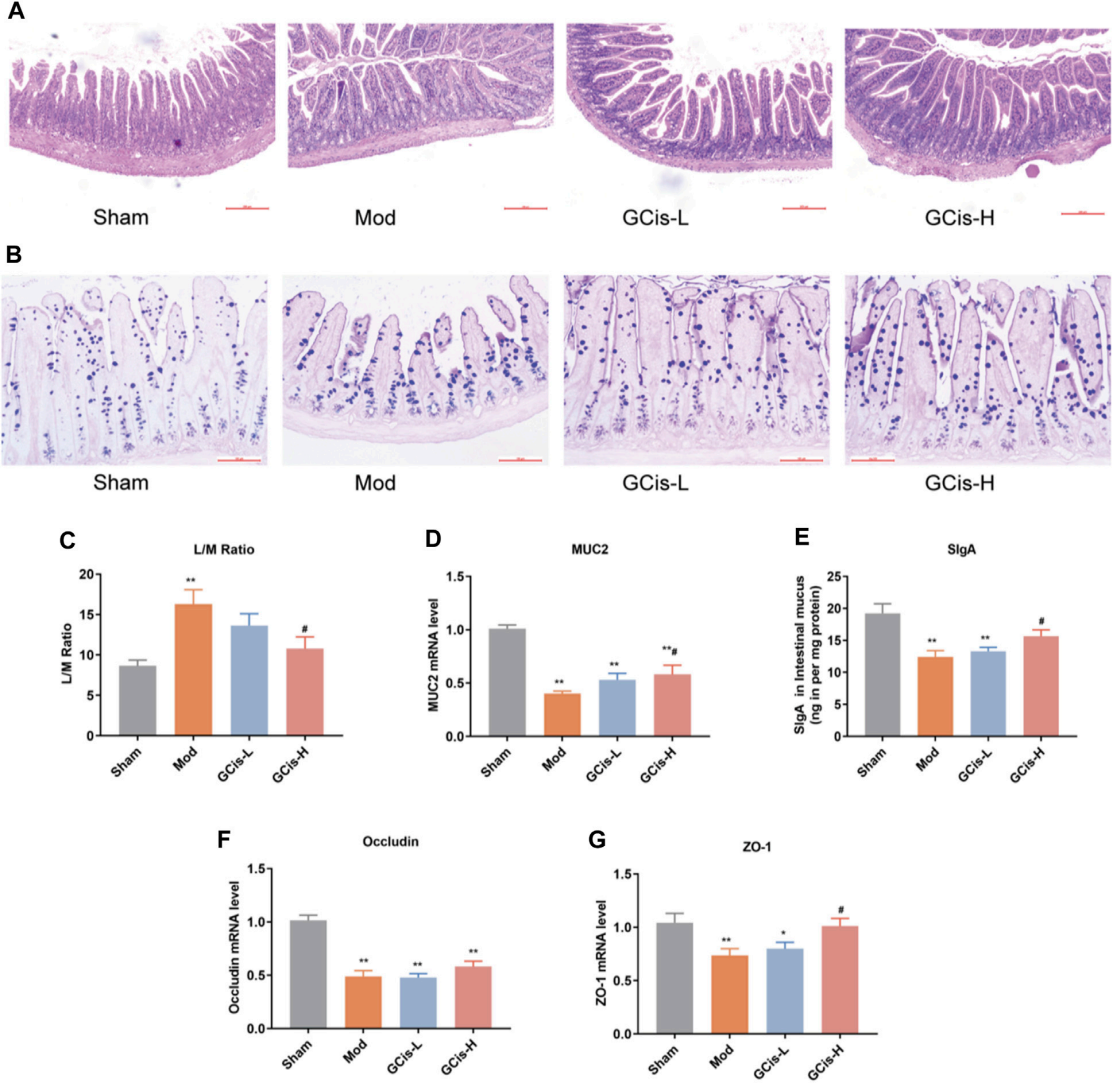
GCis formula enhanced the intestinal barrier function. HE **(A)** (magnification ×100) and AB-PAS **(B)** (magnification ×200) staining of ileum tissue. **(C)** Intestinal permeability was evaluated by L/M. MUC2 **(D)** and SlgA **(E)** were detected by ELISA. The expression of key indicators of the intestinal mucosal barrier occluding **(F)** and ZO-1 **(G)** (data are shown as mean ± SEM. **p < 0.01 compared with the sham group; ^#^ *p* < 0.05 compared with the model group).

In the model group, HE and AB-PAS staining of ileum tissue showed edema in the lamina propria and mucosal layer of the ileum compared with the sham group. Moreover, an obvious reduction of goblet cells in the mucosal layer was observed in the model group. Following the GCis formula, the edema in the lamina propria and the mucosal layer was significantly improved, and the goblet cells in the mucosal layer were increased (Figures 5A,B).

L/M evaluated intestinal permeability in the ileum tissue of the ICH model mice. In this study, the expression levels of L/M in the ileum tissue were significantly higher in the model group than in the sham group (*p* < 0.01). The GCis formula treatment significantly reduced the expression levels of L/M in the ileum tissue relative to the model group (*p* < 0.05, Figure 5C). MUC2 and SlgA were significantly lower in the model group than in the sham group (*p* < 0.01) but higher than that of the model group following the GCis formula (*p* < 0.01, Figures 5D,E).

The expression of key indicators of the intestinal mucosal barrier (occludin and ZO-1) in ileum tissues was significantly lower in the model group than in the sham group (*p* < 0.01). ZO-1 was significantly higher in the GCis formula group than in the model group (*p* < 0.05), and occludin showed no significant difference in the GCis formula group and model group (Figures 5F,G). These results indicate that the GCis formula improved the intestinal barrier function.

#### Transcriptome Analysis of DEGs

**FIGURE 6:**
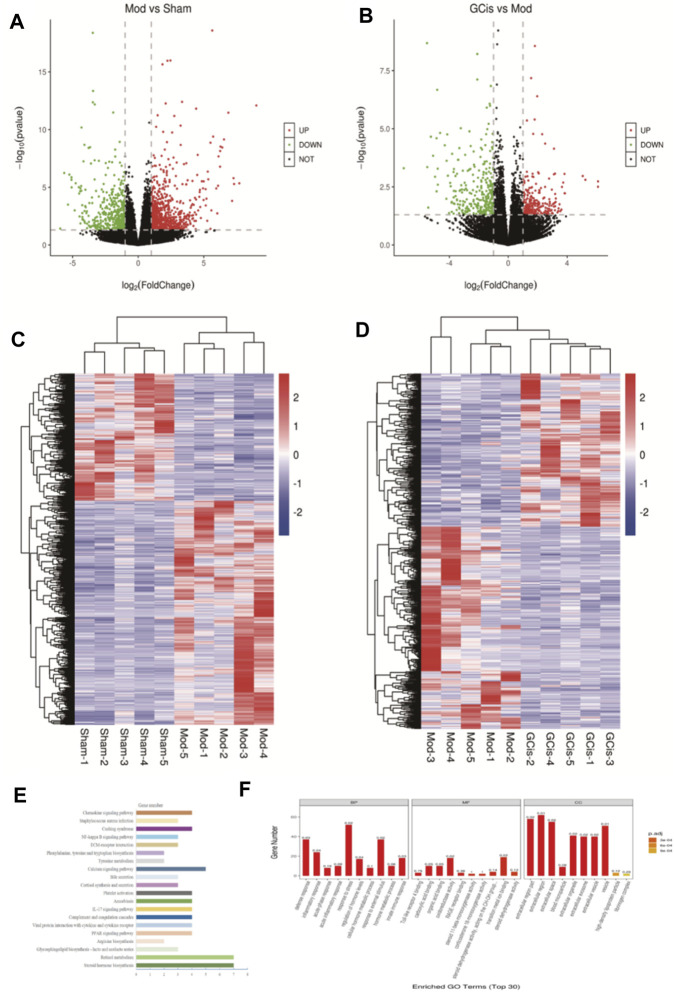
Transcriptome analysis of DEGs in the ileum. The upregulated (red) or downregulated (blue) genes **(A)**. **(C)** Sham group *vs*. model group **(B)**. **(D)** GCis group *vs*. model group. Bioinformatics analysis (GO and KEGG annotation) of 228 DEGs. **(E)** GO analysis of GCis 228 DEGs. **(F)** KEGG analysis of GCis 228 DEGs.

DEGs were screened out by *p*-value ≤ 0.05 and |log_2_ foldchange|≥1. The volcano plot showed the relative changes in gene levels (Figures 6A,B). Compared with the sham group, 1,281 differentially expressed genes were screened out in the model group, including 817 upregulated genes and 464 downregulated genes. A total of 555 differentially expressed genes were screened out between the GCis formula group and the model group, including 239 upregulated genes and 316 downregulated genes. The hierarchical cluster was used to obtain the sample clustering results (Figures 6C,D). There were significant differences in gene expression between the model and sham groups and the GCis and model groups and high similarity in gene expression between the same groups.

We screened 228 differentially expressed genes after GCis formula intervention, including 66 upregulated genes and 162 downregulated genes. The GO and KEGG analyses were further performed on the differentially regulated genes so that the differentially expressed genes were significantly correlated with biological functions, biochemical metabolic pathways, and signal transduction pathways (Figures 6E,F). Combined with the pathways enriched by regulated differential genes after GCis formula treatment, the two same pathways ranked first: chemokine signaling pathway, NF-κB signaling pathway, platelet activation, and amebiasis signaling pathway.

#### Validation of DEGs

**FIGURE 7:**
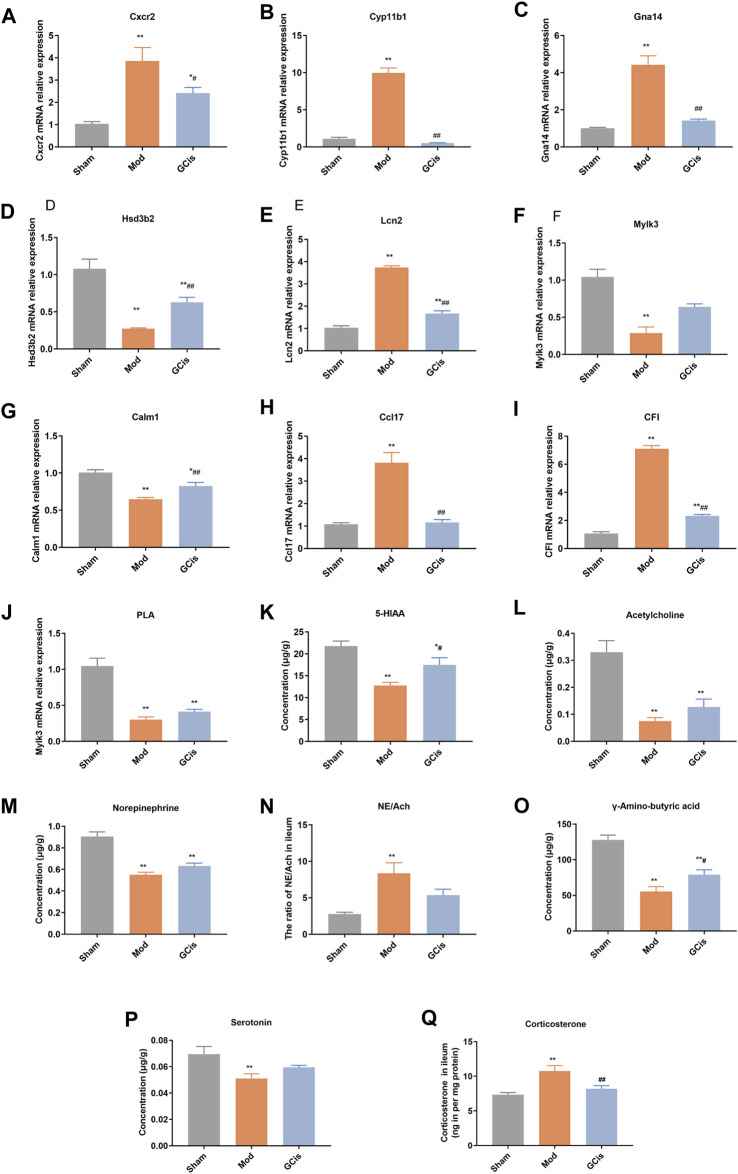
Validation of Cxcr2 **(A)**, Cyp11b1**(B)**, Gna14 **(C)**, Hsd3b2 **(D)**, Lcn2 **(E)**, Mylk3 **(F)**, Calm1 **(G)**, Ccl17 **(H)**, CFI **(I)**, PLA **(J)**, 5-HIAA **(K)**, acetylcholine **(L)**, norepinephrine **(M)**, NE/Ach **(N)**, γ-amino-butyric acid **(O)**, serotonin **(P)**, and corticosterone **(Q)** (data are shown as mean ± SEM. **p < 0.01 compared with the sham group; ^#^ *p* < 0.05 and ^##^ *p* < 0.01 and compared with the model group).

To further verify the accuracy of sequencing results, qRT-PCR was used to detect the expression of genes in the intestinal barrier function-related pathways in ileum tissue. Compared with the sham group, the mRNA levels of Cxcr2, Cyp11b1, Gna14, Lcn2, Ccl17, and CFI in the model group were significantly upregulated (*p* < 0.01). However, the mRNA levels of Hsd3b2, Mylk3, Calm1, and PLA were significantly downregulated (*p* < 0.01, Figures 7A–J). Compared with the model group, the expression levels of Cxcr2, Cyp11b1, Gna14, Lcn2, Ccl17, and CFI mRNA in the GCis formula group were significantly downregulated (*p* < 0.01 and *p* < 0.05), and the expression levels of Hsd3b2 and Calm1 mRNA were upregulated (Calm1). In contrast, Mylk3 and PLA showed no significant difference after GCis formula treatment. Cxcr2, Ccl17, Lcn2, and other genes played important roles in regulating immune, inflammatory, and antimicrobial responses. PLA, Calm1, and Mylk3 are key targets of the platelet activation pathway and play significant roles in regulating inflammatory responses. These results indicate that the GCis formula may inhibit the expression of Cxcr2, Ccl17, and Lcn2; promote the expression of PLA, Calm1, and Mylk3; and participate in the regulation of chemokine signaling pathway, platelet activation, and amebiasis signaling pathway to improve the intestinal barrier function.

The hypothalamic-pituitary-adrenal (HPA) axis, sympathetic nervous system, and parasympathetic nervous system may cooperatively mediate immune function and intestinal barrier function after CNS injury (Stanley et al., 2016; Prüss et al., 2017; Zhang et al., 2018). The pathways were mainly related to some neurotransmitters. Compared to the sham group, 5-hiAA (Figure 7K), acetylcholine (Figure 7L), Norepinephrine (Figure 7M), gamma-amino-butyric acid (Figure 7O), and serotonin (Figure 7P) of model group were significantly decreased (*p* < 0.01), while 5-HIAA and γ-amino-butyric acid were significantly increased with GCis treatment (*p* < 0.05). A memorably higher level of NE/Ach (Figure 7N) and corticosterone (Figure 7Q) were observed in the model group (*p* < 0.01), while corticosterone was significantly decreased with the GCis treatment (*p* < 0.01).

## Discussion

Clinical and experimental studies have shown that lung infection is a common complication induced by stroke and seriously affects the prognosis and life quality of patients (Chen et al., 2017; Hoffmann et al., 2017; Kigerl et al., 2018; Saand et al., 2019). Types of bacteria that have been commonly detected in the sputum and urine of stroke patients are common commensal bacteria that reside in the human intestinal tract (e.g., *Enterococcus* spp. and *Escherichia coli*). Peripheral immunosuppression and intestinal barrier dysfunction are key driving factors in its pathological progression (Mebius et al., 2005; Caso et al., 2016; Malone et al., 2019). For this, an effective and key means for lung infection is antimicrobial therapy (Lin et al., 2015; Westendorp et al., 2015). However, in clinical practice, effective therapeutic strategies are still urgent (Ma and Li., 2020). Therefore, in the present study, using collagenase IV-S-induced ICH mice, we attempted to demonstrate the therapeutic activities of the GCis formula, an extract from TCM including Renshen (White Ginseng), Fuzi (Hei Shunpian), and Rou Congrong combination (1:1:1). 16S/RPP30 and inflammatory changes in the lung tissue are common symptoms of lung infection. The results presented showed that the GCis formula prevented pulmonary infection induced by ICH, indicating a potential therapeutic capability for a lung infection, and had no effect on inflammatory factors in the brain.

In lung infection, inflammatory cytokines play prominent roles, while being important, which might be notably inhibited with GCis treatment. TNF-α, IL-6, and IL-1β induce an accumulation of inflammatory cells, provoking the generation of inflammatory mediators (Malaviya et al., 2017; Shibata et al., 2018). Mip-1α and MCP-1 are chemotactic cytokines that play an important role in promoting the circulation of effector cells to inflammatory sites (Brandenberger et al., 2018; Yang et al., 2020a). For these inflammatory mediators, GCis treatment memorably decreased their production in lung tissue of model mice, further strongly demonstrating its potential therapeutic property on lung infection.

Furthermore, potential mechanisms of the GCis formula on pulmonary infection induced by ICH were discussed here. Previously, it was found that peripheral immunity and intestinal mucosal immune barrier played critical roles in pulmonary infection (Camara-Lemarroy et al., 2014; Houlden et al., 2016).

Immunosuppression of IV-S-induced ICH mice mainly showed loss of lymphocyte, splenic contraction, and an increase in anti-inflammatory cytokine (Planas et al., 2012). The spleen, the largest secondary lymphoid organ, is the main reservoir of peripheral immune cells, combining innate and adaptive immune systems in a unique way (Pennypacker, 2014). Transcriptome focuses on the expression of functional genes and the molecular mechanism of biology, which has become a relatively mature research method in the field of biology. RNA-seq high-throughput sequencing technology has been successfully applied in studying various diseases, drugs, and biology. It can explain the functional characteristics of “multi-component and multi-target” TCM from the gene level, which is conducive to the modernization of TCM (Xu et al., 2016; Li et al., 2017). In this study, the results showed that the spleen and thymus index of the model group were all notably enhanced with the GCis treatment. Our transcriptomics data showed that GCis controlled 420 DEGs. Bioinformatics analysis suggested that the signaling pathways affected by GCis contained synthesis and release of immune cells, B-cell-mediated immunity, lymphocyte-mediated immunity, adaptive immune response based on somatic recombination of immune, and Toll-like receptor signaling pathway (Fu et al., 2015). H2-Q7, Cd209a, Ccr6, NF-κB1, PI3K, and CR2 are important targets that regulate immune, inflammatory, and antimicrobial responses (Hop et al., 2017).

Effective improvement of the intestinal barrier function is the key to alleviating pulmonary infection, such as intestinal permeability and intestinal mucosal immune (Manga et al., 2009; Metidji et al., 2018). Our present study also showed intestinal barrier injury, such as infiltration of lymphocytes and monocytes in the intestinal mucosal layer in ileum tissues, and a decrease in intestinal tight junction proteins (occludin and ZO-1) of the model mice (Crapser et al., 2017). GCis treatment significantly improved intestinal barrier injury. The results of ileum transcriptomics analysis showed that the GO and KEGG pathway enrichment of reversed genes after GCis formula intervention mainly included chemokine signaling pathway, NF-κB signaling pathway, platelet activation, and amebiasis signaling pathway. Cxcr2, Ccl17, and Lcn2 were the key targets that regulate the intestinal immune (Huang et al., 2016). PLA, Calm1, and Mylk3 are key targets of the platelet activation pathway and play significant roles in regulating inflammatory responses (Wu and Liu, 2012; Courties et al., 2019). Finally, we used molecular biotechnology to verify the credibility of quantitative transcriptomics by selected genes from DEGs, including Cxcr2, Cyp11b1, Gna14, Lcn2, Ccl17, CFI, Hsd3b2, Mylk3, Calm1, and PLA (Hop et al., 2017).

Our study has several limitations. First, the method of making ICH models or the choice of collagenase dosage may affect the animal’s performance. We used a collagenase injection model and cannot rule out the possibility that the results would differ in other models of brain damage. Second, our study preliminarily evaluated the influence of stroke at different time points on immune function without a specific analysis of lymphocyte typing. However, it is a future line of investigation in the laboratory. Third, although we described a vital source of secondary infection poststroke, we did not establish bacterial translocation and dissemination after experimental induction of stroke, which will be addressed in a future study.

In conclusion, the presented study unveils the ameliorated effects of the GCis on pulmonary infection, suggesting its potential therapeutic usages in lung infection treatment partly through enhancing peripheral immunity and intestinal mucosal immune barrier. Nevertheless, because of the limited time and experimental conditions, some mechanisms were not explored. Immunoregulation has not been clearly proven. In addition to the foregoing limitations, compounds in the GCis have the complexity and diversity, and the exact effective components and whether they have pharmacodynamic synergy also need definition.

## Data Availability

The datasets presented in this study can be found in online repositories. The names of the repository/repositories and accession number(s) can be found in the article/Supplementary Material.
