# Supplementary figures and images for: Herb Formula (GCis) Prevents Pulmonary Infection Secondary to Intracerebral Hemorrhage by Enhancing Peripheral Immunity and Intestinal Mucosal Immune Barrier
Source: Front Pharmacol. 2022 May 23;13:888684. doi: 10.3389/fphar.2022.888684 (PMC9168277; doi:10.3389/fphar.2022.888684)

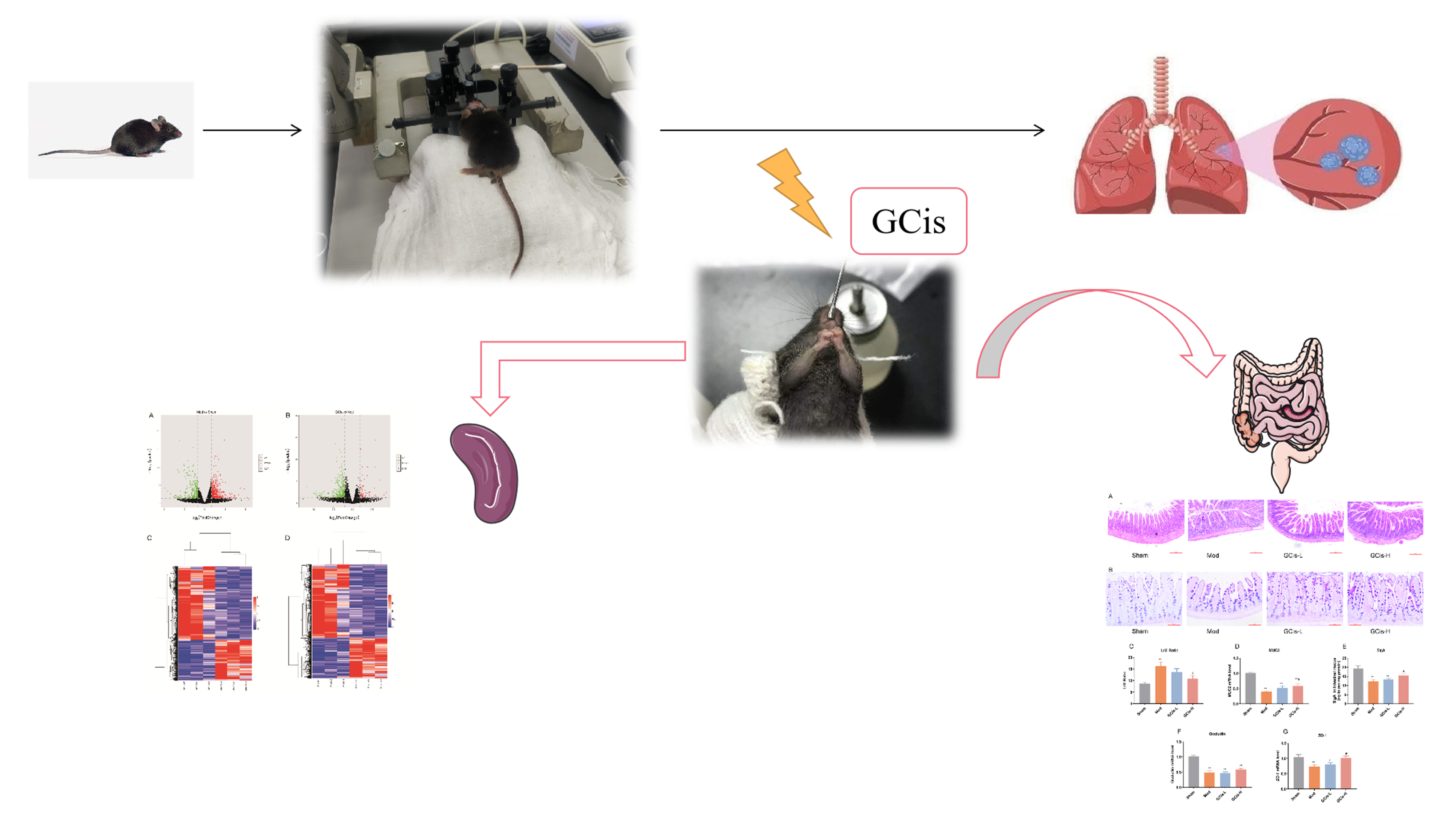

Supplement: Supplementary file 1 [file Image1.TIF]
